# 2744. A 5-Year Single-Center Review of *Strongyloides* Seropositivity in Kidney Transplant Candidates in Rural Central Texas

**DOI:** 10.1093/ofid/ofad500.2355

**Published:** 2023-11-27

**Authors:** Robin Snellings, Collin M Telchik, Sowmya Padakanti, Juan Negron-Diaz, Lizbeth Cahuayme-Zuniga

**Affiliations:** Baylor Scott & White Medical Center, Temple, Texas; Baylor Scott & White Medical Center, Temple, Texas; Baylor Scott and White Hospital, Temple, Texas; Baylor Scott & White Medical Center, Temple, Texas; University of Michigan, Ann Arbor, Michigan

## Abstract

**Background:**

The American Society of Transplantation recommends screening for *Strongyloides stercoralis* for people from or with extended travel to endemic areas including tropical regions of Southeast Asia and the Appalachian region of the United States. Asymptomatic carriers without risk factors can potentially be missed during screening assessments. Consequently, they can develop deadly hyperinfection and disseminated disease with immunosuppressive therapy. The aim of this study is to identify demographics and characteristics among our population of *Strongyloides*-infected kidney transplant candidates in rural Central Texas.

**Methods:**

This study is a single-center, retrospective chart review of patients aged 18 or older who underwent evaluation for kidney transplant at our institution in rural Central Texas between January 1, 2018 and December 31, 2022 and tested equivocal or positive for serum *Strongyloides* IgG antibody.

**Results:**

Out of the 1653 patients who underwent *Strongyloides* screening during their evaluation for kidney transplant within the study period, 182 (11.0%) returned as either equivocal or positive. Demographics of this *Strongyloides*-infected population are noted in table 1. 86.3% of these patients were born in the United States or Canada, 6.6% from Mexico, and 3.3% from Asia. 33.0% reported no international travel outside the United States (table 1). Comorbidities and symptoms of our population are listed in table 2. 83.5% were asymptomatic. Laboratory data and other risk factors for *Strongyloides* infection are noted in table 3. Only 6.0% had eosinophilia at time of diagnosis (considered as greater than 0.00 - 0.76 10*9/L). None of our population tested positive for human T-lymphotrophic virus (HTLV-1). Out of the 69 patients who were asked, 71.0% reported a history of walking outside barefoot.

**Figure:**
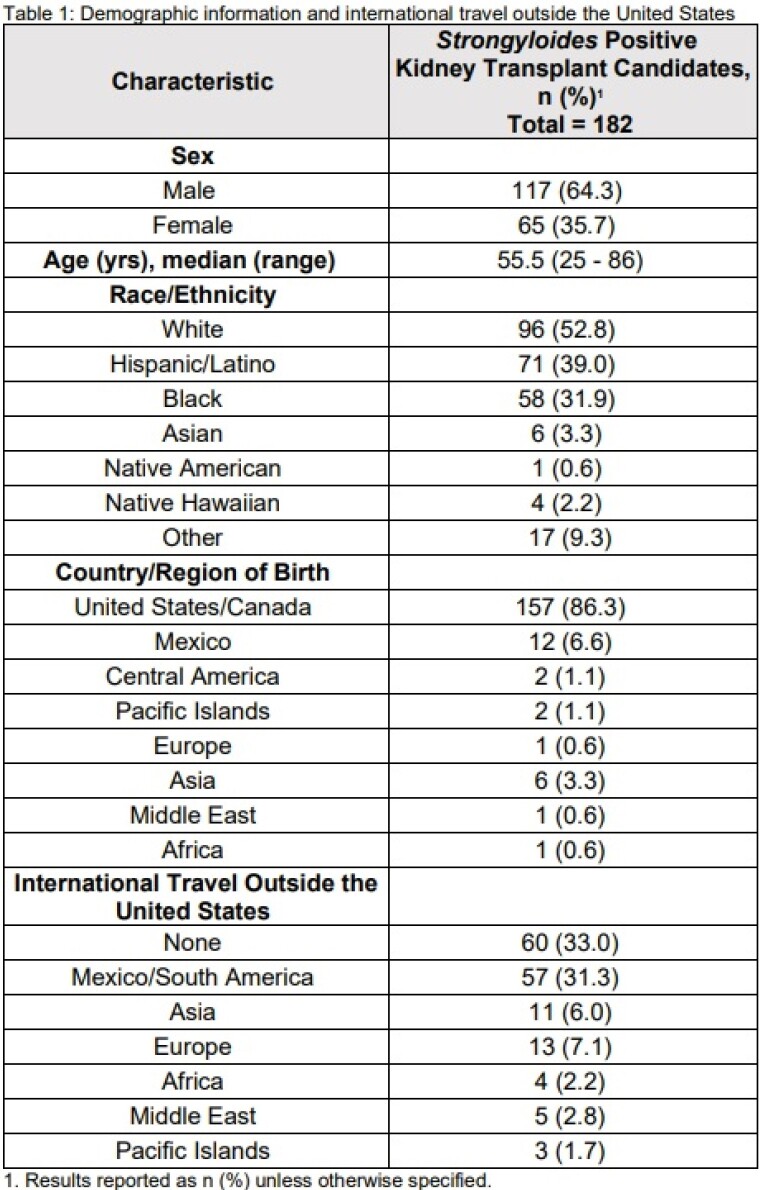

Demographic information and international travel outside the United States

**Figure:**
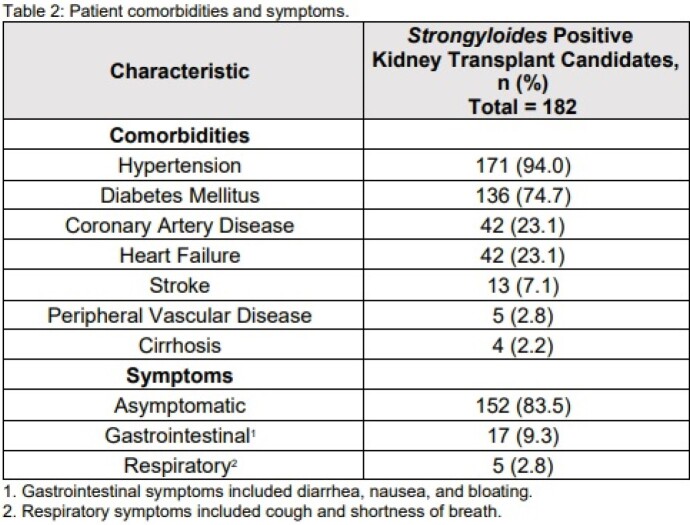

Patient comorbidities and symptoms

**Figure:**
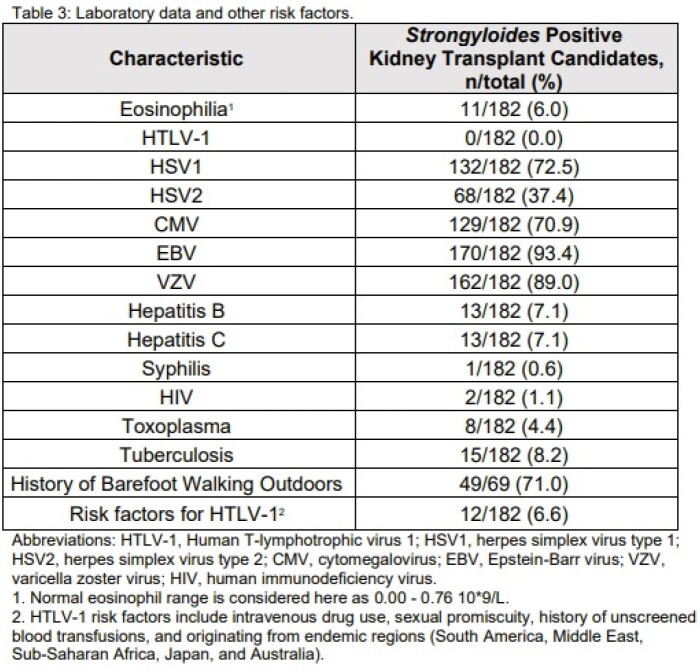

Laboratory data and other risk factors

**Conclusion:**

Importantly, 11.0% of our population were found to have an equivocal or positive *Strongyloides* serology. Of those, 33.0% had no apparent risk factors for *Strongyloides* infection including international travel, suggesting local acquisition of the infection in rural Central Texas. More research is needed to identify unique risk factors for this population including environmental studies of this underserved population.

**Disclosures:**

**All Authors**: No reported disclosures

